# Which Life Style Anthropometric Index is a Better Predictor of Renal Function?

**DOI:** 10.5812/ijhrba.6708

**Published:** 2012-11-26

**Authors:** Mansour Shahraki, Touran Shahraki, Faramarz Fazeli, Hoshang Sanadgol

**Affiliations:** 1 Department of Nutrition, Faculty of Medicine, Zahedan University of Medical Sciences, Zahedan, IR Iran; 2 Children and Adolescent Health Research Center, Zahedan University of Medical Sciences, Zahedan, IR Iran; 3 Department of Pediatrics, Zahedan University of Medical Sciences, Zahedan, IR Iran; 4 Department of Urology, Faculty of Medicine, Zahedan University of Medical Sciences, Zahedan, IR Iran; 5 Department of Nephrology, Faculty of Medicine, Zahedan University of Medical Sciences, Zahedan, IR Iran

**Keywords:** Prevention and Control, Life Style, Renal Insufficiency

## Abstract

**Background:**

Obesity is a risk factor that has been associated scientifically with hypertension, diabetes, hyperlipidemia, cancer and other life threatening diseases. The results of some studies have also shown that obesity is an independent risk factor for the development and progression of renal damage.

**Objectives:**

The aim of the current study is to define which general and central obesity anthropometric indices are better predictors for ceratinine clearance (CC) in healthy, normal and obese Iranian women.

**Patients and Methods:**

In this case-controlled study, a total of 62 healthy, normal and obese women from 18 to 30 years of age in Zahedan City, the Islamic Republic of Iran, were studied. The subjects were classified into two groups; case group (31 subjects) of healthy obese women (30 ≤ BMI ≤ 39.9 kg/m^2^) and control group (31 subjects) of healthy normal women (18.5 ≤ BMI ≤ 24.9 kg/m^2^). An assessment of body mass index (BMI) was considered as a general obesity index and an assessment of waist circumference (WC) and waist to hip ratio (WHR) were considered as central obesity indices. A measurement of CC was considered for renal function.

**Results:**

The means of CC in subjects with increased BMI, WC, and WHR were significantly higher than those in subjects with normal BMI, WC, and WHR. Pearson correlation coefficient revealed that there was a stronger correlation between CC with WC than with WHR and BMI (r = 0.4, P = 0.009; r = 0.4, P = 0.01 in the case and control groups, respectively).

**Conclusions:**

It is suggested that in clinical practice, WC can be used as a better predictor of CC than WHR and BMI in both normal and obese, healthy women.

## 1. Background

Obesity is a significant risk factor associated with hypertension, diabetes, hyperlipidemia, cancer and other life threatening diseases (1, 2). Results of studies have also shown that obesity is an independent risk factor for the development and progression of renal damage (3). Nowadays, because of the increasing prevalence of obesity in both developed and developing countries, it appears that obesity has become one of the most important public health concerns (4). Usually, to make an assessment of obesity, anthropometric indices such as; body mass index (BMI), waist circumference (WC), and waist to hip ratio (WHR) are used. Generalized obesity is measured by BMI, but abdominal obesity, which is closely associated with intra-abdominal fat, is measured by WC or WHR (5). There are some articles about the comparison of anthropometric indices as predictors of serum lipids (6), cardiovascular risk factors (7-9) and type two diabetes mellitus (10), but in the literature, the number of articles concerning the comparison of anthropometric indices as predictors of blood parameters of renal function is insufficient. In research conducted on elderly subjects, it has been reported that in indices of obesity, WHR is better than BMI, body weight and waist circumferences in predicting CKD (chronic kidney disease) in elderly Taiwanese subjects (11). The result of a study in Turkey has shown that the influence of BMI on kidney function is more prominent than WC and WHR (12).

Overall, not only are the results of available studies scarce and also controversial, but up to now, few studies have evaluated the effect of anthropometric indices on ceratinine clearance (CC) in normal and obese subjects. Women are at greater risk of obesity than men (13), we have therefore run our project on normal and obese, healthy women.

## 2. Objectives

The aim of the present study was to predict that which general and central anthropometric indices have more effect on CC in healthy, normal and obese women.

## 3. Patients and Methods

### 3.1. Study Subjects and Geographical Area

In this case-control study, a total of 62 normal and obese, healthy women between 18 to 30-years old were studied to determine the correlation between general and central obesity indices and CC. The study was carried out in Zahedan City. This city is the center of the Sistan and Baluchestan Province, which is situated in the southeast of Iran. Based on the World Health Organization (WHO) standards for BMI (14), the subjects were classified into two groups; case group (31 subjects) healthy obese women (30 = BMI = 39.9 kg/m^2^) who have referred to the nutrition clinic only for a checkup) and control group (31 subjects) of healthy normal women (18.5 = BMI = 24.9 kg/m^2^) who were medical students and they participated in the study as volunteers. The project was approved by the Medical Faculty Research Committee (no: 1157) of Zahedan University of Medical Sciences. Informed consent to participate in the study was obtained from the subjects.

### 3.2. Anthropometric Measurements

In this study, an assessment of BMI was considered as a general obesity index and assessment of WC and WHR were considered as central obesity indices. All anthropometric measurements were made in accordance with WHO standards (15) by trained staff during clinical visits. In women, a BMI of equal or more than 30, WC more than 88 cm and WHR more than 0.8 were accepted as increased values in general and central obesity indices (16).

### 3.3. Inclusion and Exclusion Criteria

On the basis of the classification of BMI (underweight: BMI < 18.5 kg/m^2^; normal: BMI 18.5 - 24.9 kg/m^2^; overweight: BMI 25.0 - 29.9 kg/m^2^; obese: BMI = 30 kg/m^2^) (16), women in an obese condition (case group) and women in normal condition (control group) were included in the study. Exclusion criteria were; subjects under 18 or over 30 years old, BMI (less than 18.5 kg/m^2^, between 25 and 29.9 kg/m^2^ or equal or more than 40 kg/m^2^), pregnant women, acute and chronic renal failure, hypertension, cerebrovascular accidents (CVA), ischemic heart disease (IHD), opiate users, cigarette smokers and alcoholics, infections, inflammations, diabetes mellitus type two, hyperlipidemia, thyroid function disorders, secondary obesity (due to hypothyroidism or Cushing syndrome), dietary treatment for cardiovascular risk factors, weight loss in the previous two months, and some chronic illnesses.

### 3.4. Evaluation of Creatinine Clearance

For the assessment of serum creatinine; 24 hour urinary creatinine and the gathering of 24-hour urine, the samples were processed by the Pastor Laboratory (a private laboratory in the city). After obtaining a blood sample in a sitting position, a special container was given to each participant to obtain her 24 hour urine in her home and then it was returned to the laboratory the next day. The serum levels of creatinine and 24-hour urinary creatinine of the subjects (case and control groups) were determined by using the colorimetric method (Jaffe). The 24-hour CC was calculated using the following formula; (urine creatinine × urine volume in 24-hours) / (serum creatinine × 1440).

### 3.5. Statistical Analysis

All statistical analyses were performed using a SPSS/PC statistical program (version 13 for Windows). Student’s t-test was used for a comparison of differences between the two groups. Pearson correlation coefficient (r) was used to measure the degree of association between anthropometric indices and CC. All mean values were presented as Mean ± Standard Deviation. A value of (P < 0.05) was considered as statistically significant.

## 4. Results

The subjects of the study consisted of 31 obese women and 31 age-matched, normal women served as control group. Table 1 shows the characteristics of the two groups on the basis of BMI. There were no significant differences between the case and control groups for the age variable. WC and WHR were significantly higher in the obese group compared to the normal group (95.7 ± 10.07 cm vs. 71.03 ± 8.85 cm, P < 0.005; 0.83 ± 0.11 vs. 0.7 ± 0.03, P < 0.005 for WC and WHR respectively). The Mean and Standard Deviation of CC in the subjects on the basis of BMI, WC and WHR are shown in Table 2. The means of CC in subjects with increased BMI, WC, and WHR were significantly higher than those in subjects with normal BMI, WC, and WHR. Pearson correlation coefficient was used to determine which general and central obesity anthropometric index is a better predictor of CC (Table 3).

**Table 1:** Characteristics of the Two Groups on the Basis of BMI, (n = 31)

	Mean ± SD	*P*	t	df	CI	
					Lower	Upper
**Age, y**		0.2	-1.2	60	-3.3	0.84
Case	25.3 ± 4.4					
Control	24.1 ± 3.8					
**WC**		< 0.005	-10.27	60	-29.5	-19.9
Case	95.7 ± 10.07					
Control	71.03 ± 8.85					
**WHR**		< 0.005	-6.84	60	-0.18	-0.1
Case	0.83 ± 0.11					
Control	0.7 ± 0.03					

Abbreviations: CI, confidence interval; WC, waist circumference; WHR, waist to hip ratio

**Table 2:** Mean and Standard Deviation of CC (mL/min) in Subjects on the Basis of BMI, WC and WHR

	Mean ± SD	*P*	t	CI	
				Lower	Upper
**BMI**		< 0.005	< 0.005	-21.37	-7.84
Case (n = 31)	108.32 ± 16.95				
Control (n = 31)	93.7 ± 8.1				
**WC**		< 0.005	< 0.005	-24.1	-10.4
Case (n = 21)	112.47 ± 17.5				
Control (n = 41)	95.14 ± 9.5				
**WHR**		< 0.005	< 0.005	-22.5	-7.9
Case (n = 20)	111.35 ± 18.77				
Control (n = 42)	96.09 ± 9.9				

Abbreviations: BMI, body mass index; CI, confidence interval; WC, waist circumference; WHR, waist to hip ratio; CC, ceratinine clearance

**Table 3:** Pearson Correlation Coefficient Between CC With General and Central Obesity Indices in Two Groups

	WC		WHR		BMI	
	r	*P*	r	*P*	r	*P*
**Case**	0.4	0.009	0.2	0.3	0.01	0.9
**Control**	0.4	0.01	0.2	0.1	0.1	0.3
**Total**	0.7	< 0.005	0.5	< 0.005	0.5	0.005

Abbreviations: BMI, body mass index; CC, ceratinine clearance; WC, waist circumference; WHR, waist to hip ratio

On the basis of the results shown in this table, there was a strong correlation between CC with WC (r = 0.4, P = 0.009; r = 0.4, P = 0.01 in case and control groups, respectively). There was no such correlation between CC with WHR and BMI.

## 5. Discussion

In the general population, overweight and obesity are important contributors to some life threatening diseases (diabetes mellitus, hypertension, and cardiovascular disease) and death (1, 2, 4). All of these diseases can accelerate the rate of CKD. Recently obesity itself, even in the absence of these risks can significantly increase CKD and promote its progression (17). In this study we have found that the means of CC in subjects with increased BMI, WC, and WHR were significantly higher than those in subjects with normal BMI, WC, and WHR. The results of our study have also shown that there was a strong correlation only between CC and WC rather than the other two anthropometric indices in the case and control groups. Chou et al. (11) reported that in indices of obesity, WHR is better than body mass index, body weight and waist circumferences in predicting CKD in elderly Taiwanese. On the other hand, Koc et al. (12) has shown that the influence of BMI on kidney function is more prominent than WC and WHR. Bavbek (18) in a study on healthy obese individuals showed a significant association between BMI and CKD independent of other potential mediators. Our study has shown that WC is a better discriminator of kidney function in both normal and obese healthy subjects. Obesity, physical inactivity, and smoking contribute to the risk of chronic kidney disease (19, 20). Women are more susceptible to these risk factors than men (21). Causes and the mechanism of correlation between renal damage with obesity are not well understood, but it is clear that some factors such as insulin resistance, mild inflammation (22) and the action of leptin (23) could be involved. Abdominal obesity is a cause of insulin resistance (24) and it is associated with sodium retention (25), glomerular hypertension and endothelial dysfunction (26). It must be emphasized that large abdominal adiposity is the most important predisposing factor for insulin resistance (27). Excessive fat stores are also an active production site of various inflammatory cytokines, responsible for enhanced levels of inflammation and oxidative stress with detrimental renal effects. This raises the possibility of excess weight or obesity creating potentially modifiable risk factors for the development of CKD (28). Further investigation is needed to clarify these mechanisms. The results of this study have shown that in clinical practice, WC is a better index than WHR and BMI indices for predicting CC in both normal and obese, healthy women. Some limitations should, however, be considered in the study. First, for the effect of general and central obesity indices on renal function, other blood parameters of renal function such as urinary albumin excretion rates, serum urea and albumin, glomerular filtration rates and micro albuminuria rates must be surveyed. Second, the range of age was limited and the number of participants was also low.
